# The risk of ovarian cancer in hormone replacement therapy users: a systematic review and meta-analysis

**DOI:** 10.3389/fendo.2024.1414968

**Published:** 2024-07-17

**Authors:** Hongqin Xiang, Liangying Wang, Liping Sun, Song Xu

**Affiliations:** ^1^ Department of Obstetrics and Gynecology, Tonglu First People’s Hospital, Hangzhou, China; ^2^ Department of Gynecology, Affiliated Hangzhou First People’s Hospital, School of Medicine, Westlake University, Hangzhou, Zhejiang, China

**Keywords:** ovarian cancer, cancer risk, hormone replacement therapy, systematic review, meta-analysis

## Abstract

**Background:**

With the increasing use of hormone replacement therapy (HRT), there is a need to understand its impact on the occurrence of female malignant tumors. This systematic review and meta-analysis aimed to assess the risk of ovarian cancer associated with HRT and its related risk factors.

**Methods:**

PUBMED, OVID, Embase, Cochrane, and Web of Science were searched from 1980 to April 2022 to identify studies on the risk of ovarian cancer and hormone replacement therapy. The random-effects model was used to estimate the pooled risk of HRT in ovarian cancer, both in cohort studies and case-control studies. Additionally, the analysis examined the outcomes associated with different types of estrogen plus progesterone regimens. Meta-regression and sensitive analysis were performed to evaluate the heterogeneity.

**Results:**

21 cohort studies (involving 15,313 cases and 4,564,785 participants) and 30 case-control studies (including 18,738 cases and 57,747 controls) were analyzed. The pooled risks of ovarian cancer for HRT users were 1.20 (95% confidence interval [CI] 1.01–1.44) from cohort studies and 1.13 (95%CI 1.04–1.22) from case-control studies. However, after restricting the study period to recent decades, the significant results indicating a higher risk disappeared in cohort studies conducted after 2010 and in case-control studies conducted after 2006. Furthermore, the continuous use of estrogen-progesterone replacement therapy (EPRT) was associated with a risk comparable to that of sequential use. Subgroup analysis showed that both estrogen replacement treatment (ERT) and EPRT had minor risks; The risk further increased with prolonged exposure time, particularly for durations exceeding 10 years. Additionally, serous ovarian cancer appeared to be more susceptible than other pathological types.

**Conclusion:**

The risk of ovarian cancer associated with HRT has been decreasing over time. However, ERT may increase this risk, particularly when used for an extended period. It is recommended that long-time users consider continuous EPRT as a safer alternative.

**Systematic review registration:**

www.crd.york.ac.uk/prospero/, identifier CRD42022321279.

## 1 Introduction

Ovarian cancer is known as the most lethal disease among malignant tumors affecting the female genital system. It is challenging to treat because most patients are diagnosed at a late stage. Epithelial ovarian cancer encompasses various histologic types, such as serous tumor, mucinous tumor, endometrioid tumor, clear cell tumor, and others. Among them, serous tumor is the most prevalent. Although the exact causes of ovarian cancer are not entirely clear, factors like persistent ovulation and gonadotropin stimulation are often reported as tumor pathogenic factors (1, 2). However, using oral contraceptives, pregnancy, and breastfeeding have been considered as protective factors. In addition, there are other risk factors to be aware of, such as smoking, obesity, and family history.

Hormone replacement therapy has been widely used to treat menopause syndrome in women. The main HRT regimens include estrogen alone or a combination of estrogen and progesterone. It is believed to have cardiovascular benefits and a therapeutic effect on osteoporosis. Women can experience the benefits of HRT long after the menstrual cycle has stopped. However, the optimal time to start therapy is within ten years of menopause or before the age of 60. According to the North American Menopause Society (NAMS), for women who begin hormone therapy more than 10 years after the onset of menopause or who are over 60 years old, the benefit-risk ratio is less favorable due to the higher absolute risks of coronary heart disease, stroke, venous thromboembolism, and dementia (3). Initiating HRT in mid-life may protect against cognitive impairment, whereas starting it in late-life could have deleterious effects (4).

However, the Women’s Health Initiative (WHI) study found a higher hazard ratio (HR) of 1.58 for invasive ovarian cancer in users of estrogen plus progestin compared to the control group (5). Some epidemiological studies have found a significant link between HRT and the risk of female cancers, especially breast cancer. However, when it comes to ovarian cancer, the outcomes of these studies have been conflicting. Some researchers have reported an increased risk of ovarian cancer associated with postmenopausal hormone use (6–12), while other studies have found contradictory results (13).

Beyond that, three meta-analyses have also reported conflicting findings regarding the risk of HRT and ovarian cancer (14–16). Therefore, we conducted this current meta-analysis to further investigate the potential association between hormone use and ovarian cancer, taking into consideration the period of research, specific type of hormone and duration of use. Moreover, we aim to examine whether certain histological subtypes of ovarian cancer are more susceptible to being influenced by hormone use.

## 2 Methods

This study adhered to the guidelines of Preferred Reporting Items for Systematic Reviews and Meta-Analyses (PRISMA). Our registration number in the CRD is 42022321279.

### 2.1 Literature search strategy

Literature from databases including PUBMED, OVID, Embase, Cochrane, and Web of Science published after 1980 was searched to identify relevant studies. We conducted a search using keywords such as “ hormone replacement therapy”, “estrogen replacement therapy” and “non-contracept hormones” in combination with “ovarian cancer” and “ovarian tumor”.

We excluded unrelated studies by checking their title and abstract. Then, we carefully reviewed the remaining articles to ensure they were relevant to our analysis. Finally, we manually scanned the references of previous review articles and meta-analyses to identify any additional published studies.

### 2.2 Study inclusion criteria

Each research has been individually checked and reviewed by three authors (HQ.X, LY.W, LP.S). In case of any conflicts, a group discussion would be conducted to resolve them. Our primary screening was based on the titles and abstracts of the research papers.

All the studies included in our analysis should meet the following criteria: (1) prospective or compared retrospective studies. (2) contain data on HRT use and ovarian cancer incidence confirmed by pathological examination. (3) the index of survival analysis, such as relative risk (RR) or odds ratio (OR), along with their corresponding 95% confidence intervals (CI), should be available.

Studies were excluded if the patients did not have a history of malignant ovarian tumor, as well as duplicate data or repeat analysis.

### 2.3 Data extraction

Two authors independently extracted risk data from these studies and conducted our meta-analysis. We also collected additional information such as author, year of publication, country, patients’ characteristics, type and duration of hormone use, cancer type, and adjusted variables in the analysis. The majority of patients had epithelial ovarian cancer, including subtypes such as serous, mucinous, endometrioid, and others.

The article would combine the result to calculate the use of ERT and EPRT based on the provided OR or RR value. If the hormone species were available, the risk estimates would be evaluated based on their grouping. The methods of HRT use were categorized as ERT and EPRT, further classified as continuous or sequential use. The adjusted estimate would be given preference over the unadjusted OR and RR. There were no restrictions on the length of follow-up. Any conflicts in this process would be resolved through group discussion.

### 2.4 Statistical analysis

We performed this meta-analysis to investigate the correlation between HRT use and the risk of ovarian cancer. We obtained a pooled risk estimate by statistically analyzing the data extracted from each individual study. The results were combined by the DerSimonian and Laird random-effects model.

We assessed the association between HRT use and ovarian cancer risk by comparing patients who had ever used it to those who had never used it. Consider the evolution of HRT regimens in recent years, we specifically analyzed the pooled RR of cohort studies conducted after 2010 and the pooled OR of case-control studies conducted after 2006. We used a random-effects model for analysis and combined the adjusted OR or RR with 95% CIs. To examine the homogeneity of the studies, we used the I2 statistics. The statistically significant heterogeneity was noted when the I2 value exceeded 50% or the P value of Q statistics was less than 0.10. We set the significance level at 0.10 to avoid type II errors.

Sensitivity analyses were conducted to evaluate publication bias using Egger’s regression asymmetry test, considering a P-value less than 0.10 as statistically significant. The results were presented with forest plots, showing each outcome as proportions with 95% confidence intervals (CI). Funnel plot asymmetry was used to assess publication bias, and all procedures were carried out using Stata 12.0 software (STATA Corporation, College Station, TX).

## 3 Results

### 3.1 Literature search results

Our primary search policy yielded a total of 4996 citations. We carefully selected the studies that focused on HRT and ovarian cancer by reading their abstracts. After a thorough review, we identified 72 articles that met our criteria. Out of these, we excluded 21 articles for reasons such as: lack of statistical data necessary for calculations, non-compliance with our research design, and outdated versions of the studies.

**Figure 1:**
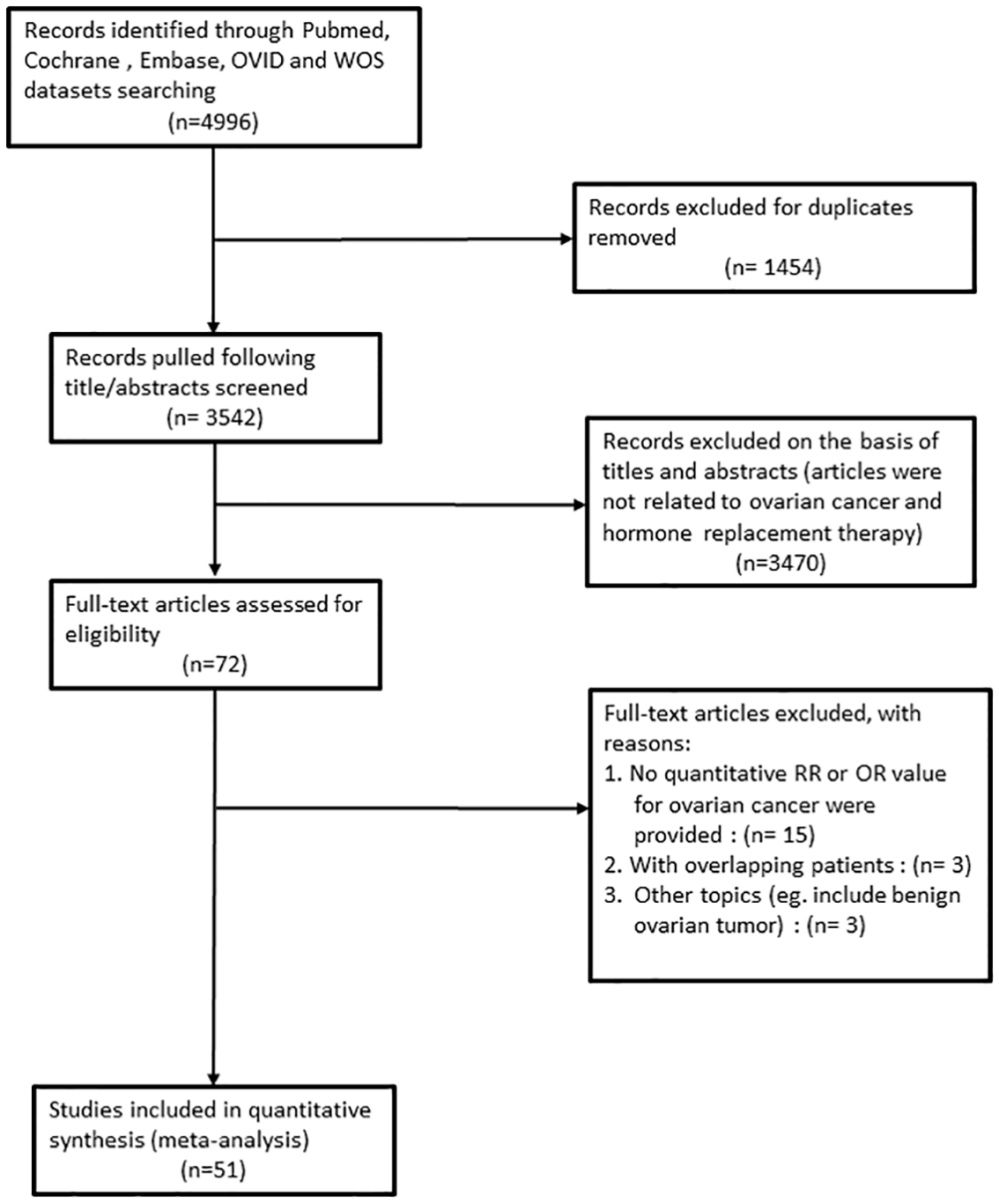
Flowchart of the study selection process according to PRISMA guidelines in this meta-analysis.

Ultimately, we found 21 cohort studies (5–7, 9, 10, 17–32) and 30 case-control studies (8, 11, 12, 33–59). The selection and exclusion process of PRISMA flow diagram is shown in **Figure 1**. Based on the Newcastle-Ottawa Quality Assessment Form, each study got a moderate or high-quality score. The analysis of different designs as cohort studies and case-control studies is as follows.

### 3.2 Characteristics of studies

In total, there were 4,564,785 participants and 15,313 patients included in 21 cohort studies. The characteristics of the studies are presented in **Table 1**. The research was conducted in Israel (n=1), several European countries (n=9), and the United States of America (n=11). The participants’ mean age ranged from 25 years to 79 years, and the average follow-up period spanned from 5 years to 26 years. We included thirty case-control studies, which involved 18,738 patients and 57,747 controls. **Table 2** shows their characteristics. These studies were conducted in various countries: Australia (n=1), Canada (n=1), China (n=1), Europe (n=11), Mexico (n=1), and the United States of America (n=15). The mean age of the participants ranged from 20 to 79 years, with an average follow-up period of 1 to 13 years.

**Table 1:** Characteristics of cohort studies included in the meta-analysis.

Study	Year	Study region	No. of Participants And cases	Follow-up (years)	Age (years)	Hormone type	RR	95% CI	Adjusted Variables
Adami et al. (15)	1989	Sweden	23244 (64)	6.7 years	54.5 (mean)	ERT	0.96	0.74, 1.23	NA
Rodriguez et al. (16)	2001	USA	211581 (944)	14 years	PMP	ERT	1.23	1.06, 1.43	Age at baseline, race, duration of OC use, number of live births, age at menopause, BMI, age at menarche, tubal ligation
Lacey et al. (7)	2002	USA	44241 (329)	13.4 years	56.6 (mean)	EPRT	1.10	0.64, 1.70	Age, menopause type, and duration of oral contraceptive use
Anderson et al. (3)	2003	USA	16608 (32)	5 years	50-79	EPRT	1.64	0.78, 3.45	age and randomization to the WHI dietary trial
Bakken et al. (17)	2004	Norway	35456 (129)	7 years	53.0 (mean), 45-64	HRT	1.30	0.80, 2.00	Age, BMI, smoking, ever use of OCs, time since menopause, parity and age at last birth
Folsom et al. (18)	2004	USA	31381 (223)	15 years	55-69	ERT	1.07	0.77, 1.50	Age, family history of ovarian cancer in a first-or second-degree relative, hysterectomy, unilateral oophorectomy, number of live births, physical activity index, pack-years of smoking, waist/hip ratio, and BMI
Kumle et al. (19)	2004	Norway and Sweden	103551 (214)	9years	Norway, 34-49, Swedish, 30-49	HRT	1.50	0.90, 2.50	Age
Kiani et al. (20)	2006	USA	13281 (71)	16 years	≥25	HRT	3.04	1.55, 5.97	Age
Lacey et al. (8)	2006	USA	97638 (214)	5 years	50–71	ERT	1.33	0.89, 2.00	Age, race, menopausal status, OC use, BMI
Beral et al. (4)	2007	United Kingdom	948576 (2273)	5.3 years	57.2 ± 4.6	HRT	1.20	1.09, 1.32	Region of residence, socioeconomic group, time since menopause, parity, BMI, alcohol consumption, and use of oral contraceptives
Danforth et al. (21)	2007	USA	82905 (389)	26 years	61.2 (mean), 30-55	HRT	1.00	0.77, 1.31	Age, parity, duration of oral contraceptive use, tubal ligation, age at natural menopause, age at menarche
Mørch et al. (22)	2009	Denmark	909946 (2681)	8 years	50-79	HRT	1.15	1.01, 1.30	Age, period of use, number of births, hysterectomy, sterilization, unilateral oophorectomy or salpingo-oophorectomy, endometriosis, infertility, and educational status
Braem et al. (23)	2010	Netherlands	2706 (375)	16.3 years	Case: 62.0 ± 4.3, control: 61.5 ± 4.6	HRT	0.97	0.69, 1.37	Age, parity, duration of OC and HRT use
Trabert et al. (24)	2012	USA	92601 (426)	10 years	50-71	EPRT	1.43	1.09, 1.86	age, race, parity, duration of oral contraceptive use, and body mass index
Yang et al. (25)	2012	USA	168323 (849)	Case: 5.1 years; control: 9.8 years	Case: 62.8 ± 5.3, control: 61.8 ± 5.4	HRT	1.33	1.16, 1.53	Age, oral contraceptive use, parity, menopausal hormone therapy
Li et al. (26)	2015	10 European countries	202206 (791)	11.7 years	52.4 (mean), 45.0-77.8	HRT	1.09	0.92, 1.30	Menopausal status, age at menopause, age at menarche, number of full-term pregnancies (FTPs), age at first FTP, duration of breast-feeding, number of miscarriages, unilateral ovariectomy, hysterectomy, HRT, OC use, IUD use, BMI, smoking status, alcohol consumption, and pre-existing diabetes
Perri et al. (27)	2015	Israel	1073 (175)	18 years	Case: 53.6 ± 10.3, control: 49.1 ± 13.4	HRT	1.98	1.21, 3.25	Mutation type, age at menarche, oral contraceptive use, parity, age at first pregnancy
Urban et al. (28)	2015	USA	74786 (461)	12.3 years	50-79	HRT	1.50	1.23, 1.83	Age and race
Bethea et al. (29)	2017	USA	59000 (115)	18 years	37.8 ± 10.3, 21-69	EPRT	1.37	0.73, 2.55	Age, questionnaire cycle, parity, lactation, age at first birth, age at last birth, hysterectomy, tubal ligation, oral contraceptive use, educational HRT attainment, and BMI
Simin et al. (30)	2017	Sweden	290186 (573)	7 years	≥40	HRT	1.09	1.00, 1.19	Age
Simin et al. (31)	2020	Sweden	1155496 (3985)	7 years	≥40	HRT	0.47	0.43, 0.52	hysterectomy, ever parous, thrombotic events, year of birth, smoking-related disorders, alcohol-related disorders, obesity, diabetes mellitus and osteoporosis

RR, relative risk; CI, confidence intervals; HRT, hormone replacement therapy; ERT, estrogen replacement therapy; EPRT, estrogen + progestin replacement therapy; OC, oral contraceptive; BMI, body mass index; PMP, post menopause. NA, not available.

**Table 2:** Characteristics of case–control studies included in the meta-analysis.

Study	Year	Study region	No. of controls	No. of cases	Follow-up	Age (years)	Hormone type	OR	95% CI	Adjusted Variables
Hildreth et al. (32)	1981	USA	1068	62	1977-1979	45-74	ERT	0.90	0.50, 1.60	Age
Weiss et al. (33)	1982	USA	611	205	1975–1979	50–74	ERT	1.30	0.90, 1.80	Age, hysterectomy status, residence
Cramer et al. (34)	1983	USA	215	215	1978–1981	53.2 (cases), 53.5 (controls)	ERT	1.56	0.85, 2.87	Parity
Tzonou et al. (35)	1984	Greece	188	112	1980–1981	NA	ERT	1.60	0.20, 12.55	Age, parity, age at menopause, use of exogenous estrogens
Hartge et al. (36)	1988	USA	244	203	1978–1981	20–79	ERT	0.60	0.40, 0.80	Age, race
Booth et al. (37)	1989	USA	293	156	1978–1983	52.4 (cases), 51.4 (controls)	HRT	1.50	0.90, 2.60	Age, social class
Kaufman et al. (38)	1989	USA	2030	377	1976–1985	18–69	HRT	0.70	0.20, 1.80	Age, race, religion, age at menarche, parity, menopausal status, age at menopause, region, date of interview, OC use
Polychronopoulou et al. (39)	1993	Greece	200	189	1989–1991	<75	HRT	5.73	1.07, 30.80	Age, schooling, weight before the disease, age at menarche, parity, age at first birth
Parazzini et al. (40)	1994	Italy	2503	953	1979–1992	23–74	ERT	1.60	1.20, 2.40	Age, marital status, education, nulliparity, age
Risch et al. (41)	1996	Canada	564	367	1989–1992	35–79	ERT	1.26	0.87, 1.84	Age, parity, OC use, tubal ligation, lactation, hysterectomy, family history of breast cancer
Hempling et al. (42)	1997	USA	705	470	1982–1995	54.9 (cases), 54.9 (controls)	HRT	0.80	0.50, 1.30	Age at diagnosis, parity, OC use, smoking, family history of epithelial ovarian cancer, age at menarche, menopausal status, income, location, education
Purdie et al. (43)	1999	Australia	855	793	1990–1993	18–79	HRT	1.20	0.90, 1.60	Age, education, residence, BMI, hysterectomy, tubal sterilization, talc use in perineal region, smoking, duration of OC use, parity, family history of breast or ovarian cancer
Salazar-Martinez et al. (44)	1999	Mexico	668	84	1995–1997	52.8 (cases), 54.6 (controls)	HRT	1.00	0.36, 2.70	Age, anovulatory index, smoking, diabetes, hypertension, physical activity, menopausal status, BMI
Tavani et al. (45)	2000	Italy	2758	971	1983–1991	22–74 (cases), 23-74 (controls)	HRT	1.80	1.30, 2.60	Age, area of residence
Chiaffarino et al. (46)	2001	Italy	2411	1031	1992–1999	18-79	HRT	1.10	0.80, 1.50	Age, center, education, parity, OC, family history of ovarian, breast cancer in first relatives
Bosetti et al. (47)	2001	Europe	5882	2501	1992-1999	NA	HRT	1.28	1.05, 1.56	age, socioeconomic level, parity, oral contraceptive use, menopausal status, type of menopause, age at menopause, as well as HRT use, duration of use, and time since last use
Modugno et al. (48)	2001	USA	1367	767	1994–1998	20-69	ERT	1.01	0.98, 1.05	adjusted for age, number of live births, years of oral contraceptive use, years of noncontraceptive estrogen use and months breastfed as continuous variables, tubal ligation, hysterectomy, family history of ovarian cancer, and family history of breast cancer as dichotomous variables, and ethnicity as a polychotomous variable
Riman et al. (49)	2002	Sweden	3870	653	1993–1995	50-74	HRT	1.41	1.15, 1.72	Age, parity, BMI (kg/m2), age at menopause, hysterectomy, duration of oral contraceptive use, and ever use of estrogen only and continuous estrogen–progestin categorized variables combinations as categorized variables
Sit et al. (50)	2002	USA	926	484	1994–1998	56.6 (cases), 55.7 (controls)	HRT	0.94	0.74, 1.19	Numbers of live births, family history of ovarian carcinoma, use, history of tubal ligation, and age at diagnosis
Tung et al. (51)	2003	USA	607	558	1993–1999	52.6-57.4 (cases), 55.8 (controls)	HRT	0.80	0.60, 1.10	Age, ethnicity, study site, education, pregnancy status, tubal ligation, and oral contraceptive pill use.
Glud et al. (52)	2004	Denmark	1011	338	1995–1999	35-79	EPRT	1.08	1.01, 1.16	adjusted for age (categorical), ever/never HT use, ever/never pregnant, number of pregnancies (linear), ever/never oral contraceptive
Pike et al. (10)	2004	USA	660	477	1992–1998	18–74	ERT	0.71	0.32, 1.61	Age, ethnicity, socioeconomic status, education, family history of ovarian cancer, tubal ligation, use of genital area talc, BMI, nulliparity, age at last birth, number of additional births, number of incomplete pregnancies, OC, menopausal status, age at natural menopause, age at surgical menopause, EPRT used by hysterectomized women; EPRT used by naturally menopausal women; ERT used by hysterectomized omen; ERT used by naturally menopausal women
Mills et al. (53)	2005	USA	1122	256	2000–2001	56.6 (cases), 55.0 (controls)	HRT	1.39	1.01, 1.93	Age, race/ethnicity, duration of OC use breastfeeding
Moorman et al. (54)	2005	USA	370	364	1999-2003	20-74	HRT	1.20	0.80, 1.60	Age, race, parity, tubal ligation, hysterectomy, BMI 1 year before interview, 1st degree family history of breast or ovarian cancer, breastfeeding, oral contraceptive use, and educational level
Kotsopoulos et al. (55)	2006	USA	375	162	NA	62.7 (cases), 61.2 (controls)	HRT	0.93	0.56, 1.56	Parity, OC use and country of residence
Rossing et al. (56)	2007	USA	781	561	2002–2005	47.0 (cases), 48.0 (controls)	ERT	1.30	0.90, 1.70	Age, county of residence, year of diagnosis/reference date, number of full-term pregnancies, and duration of hormonal contraception
Schneider et al. (57)	2009	United Kingdom	516	86	1987-2007	51.3 ± 6.1	HRT	0.97	0.61, 1.54	Smoking status, BMI, use of oral contraceptives, progesterone preparations and vaginal estrogens
Koskela-Niska et al. (58)	2013	Finland	11325	3958	NA	>50	ERT	0.93	0.76, 1.13	Age and place of residence
Pasalich et al. (59)	2013	China	500	500	2006-2008	59.0 ± 5.6 (cases), 59.7 ± 6.4 (controls)	HRT	1.05	0.35, 3.21	Age, smoking status, alcohol drinking, education, BMI, mutually adjusted for parity, oral contraceptive use, hormone replacement therapy, menopausal status, hysterectomy and family history of ovarian and/or breast cancer
Rasmussen et al. (60)	2017	Denmark	13122	885	1978-2002	NA	HRT	1.32	1.02, 1.72	Age, tubal ligation, salpingectomy, hysterectomy, endometriosis, pelvic inflammatory disease, infertility, parity, and hormone replacement therapy

OR, odds ratio; CI, confidence intervals; HRT, hormone replacement therapy; ERT, estrogen replacement therapy; EPRT, estrogen + progestin replacement therapy; OC, oral contraceptive; BMI, body mass index. NA, not available.

### 3.3 Risk of ovarian cancer in prospective and retrospective studies

**Figure 2:**
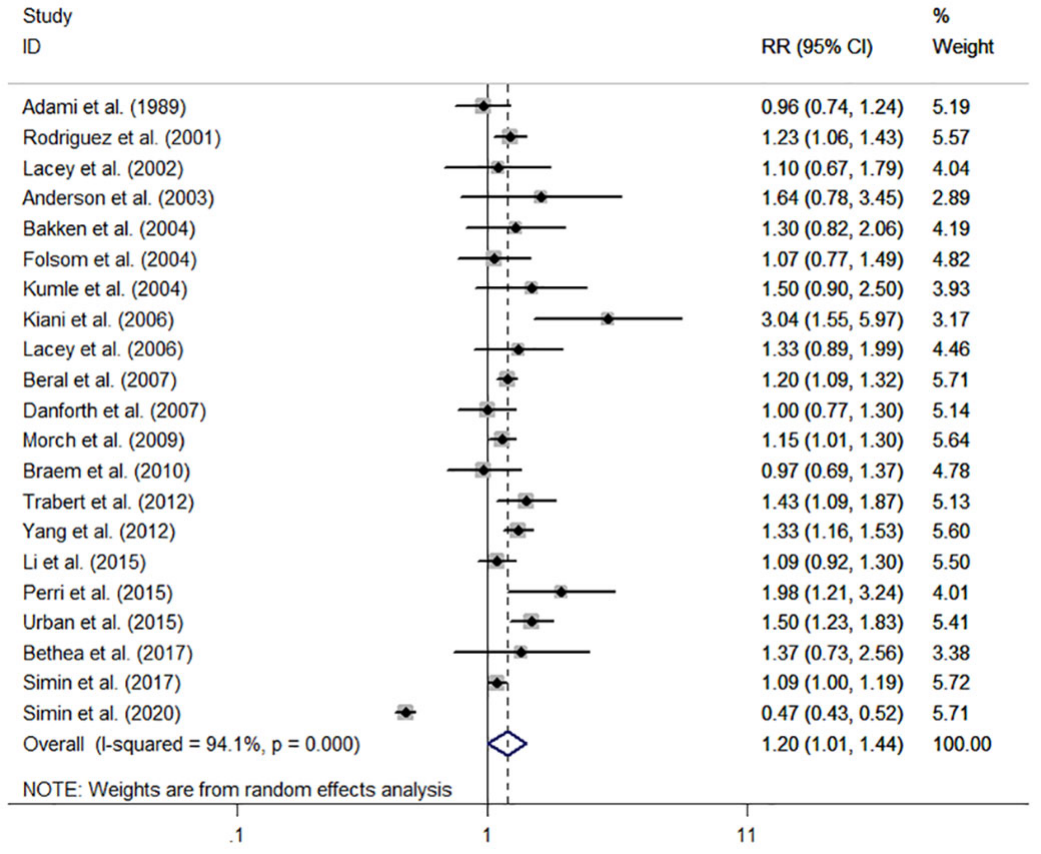
Forest plot of the association between HRT and the risk of ovarian cancer in cohort studies. The size of each gray box is proportional to the weight assigned to the respective study, with horizontal lines representing the 95% confidence intervals (CIs).

**Figure 3:**
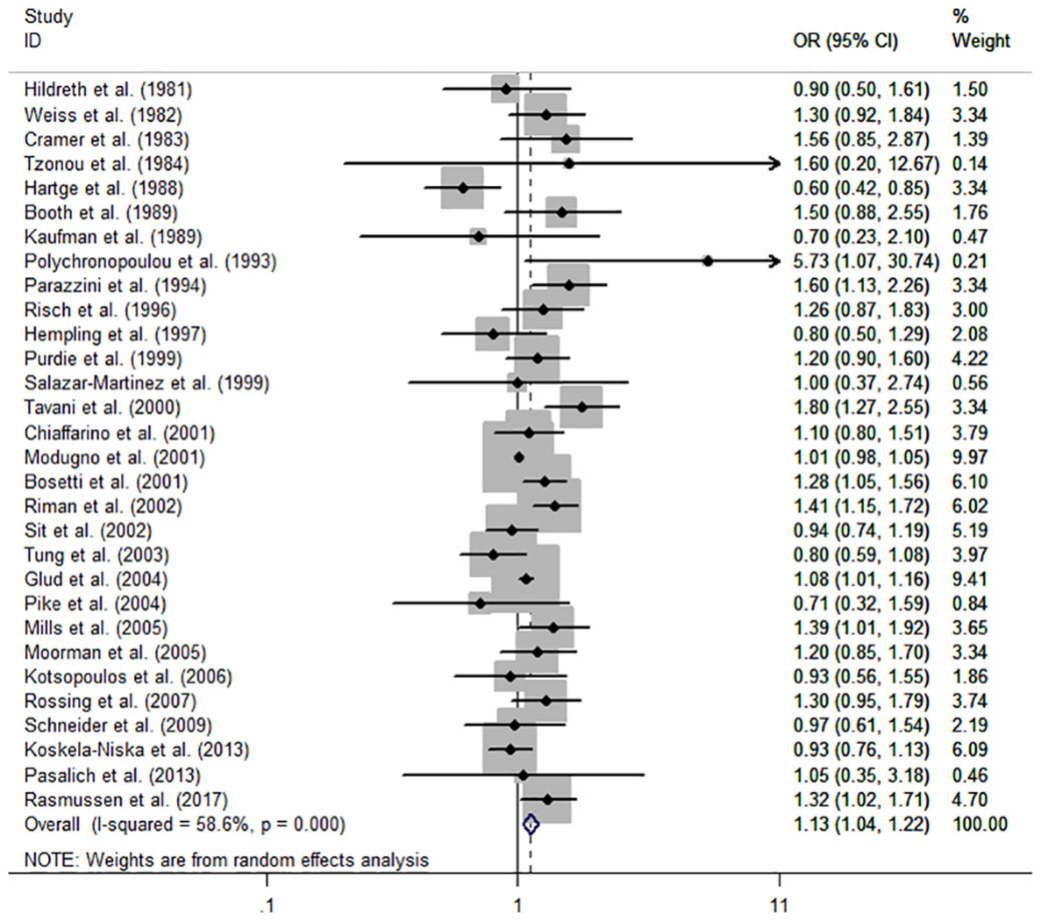
Forest plot of the association between HRT and the risk of ovarian cancer in case-control studies. The size of each gray box is proportional to the weight assigned to the respective study, with horizontal lines representing the 95% confidence intervals (CIs).

**Figure 4:**
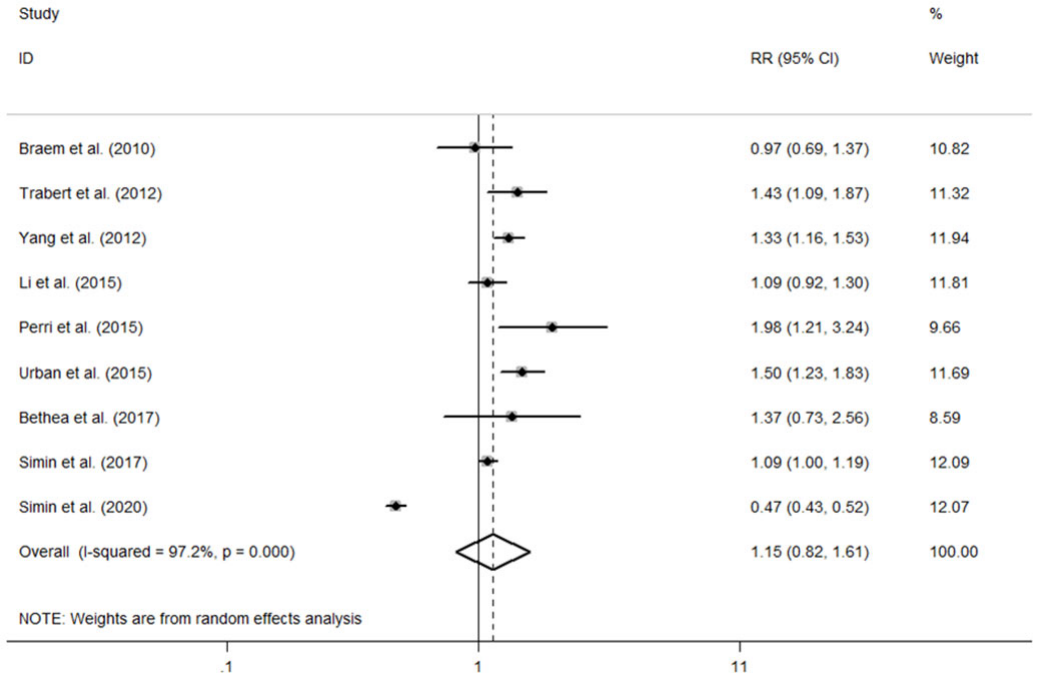
Forest plot of the association between HRT and the risk of ovarian cancer in cohort studies after 2010. The size of each gray box is proportional to the weight assigned to the respective study, with horizontal lines representing the 95% confidence intervals (CIs).

**Figure 5:**
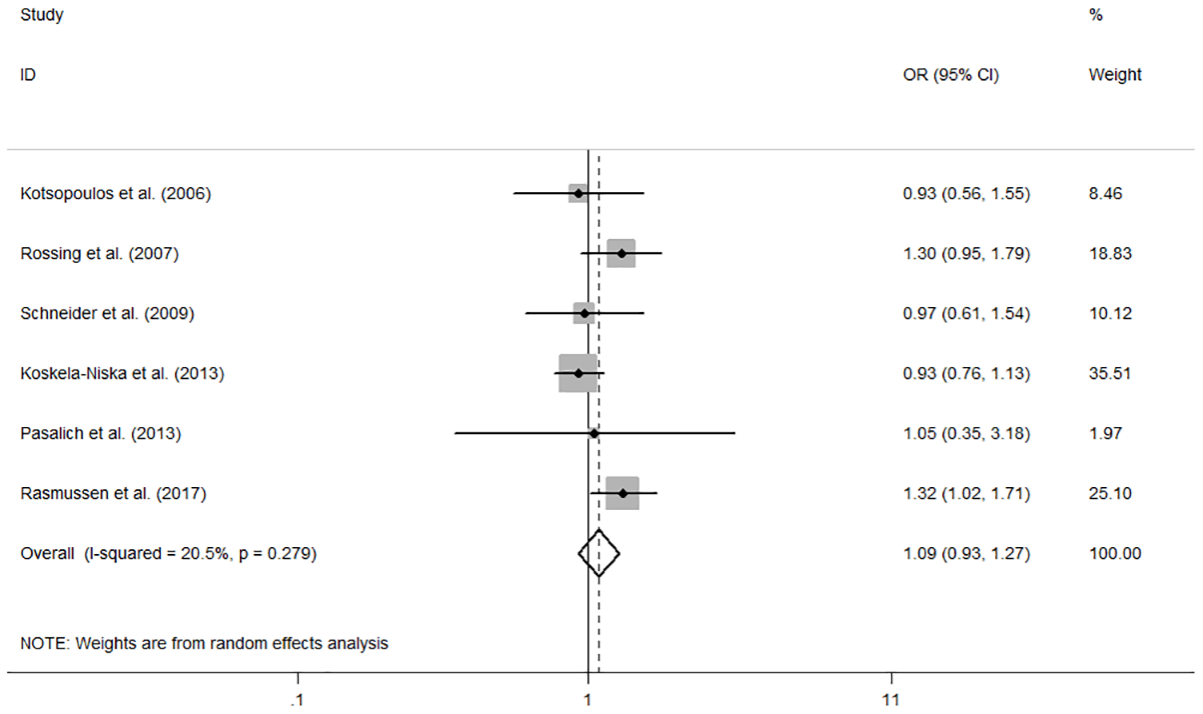
Forest plot of the association between HRT and the risk of ovarian cancer in case-control studies after 2006. The size of each gray box is proportional to the weight assigned to the respective study, with horizontal lines representing the 95% confidence intervals (CIs).

**Figure 6:**
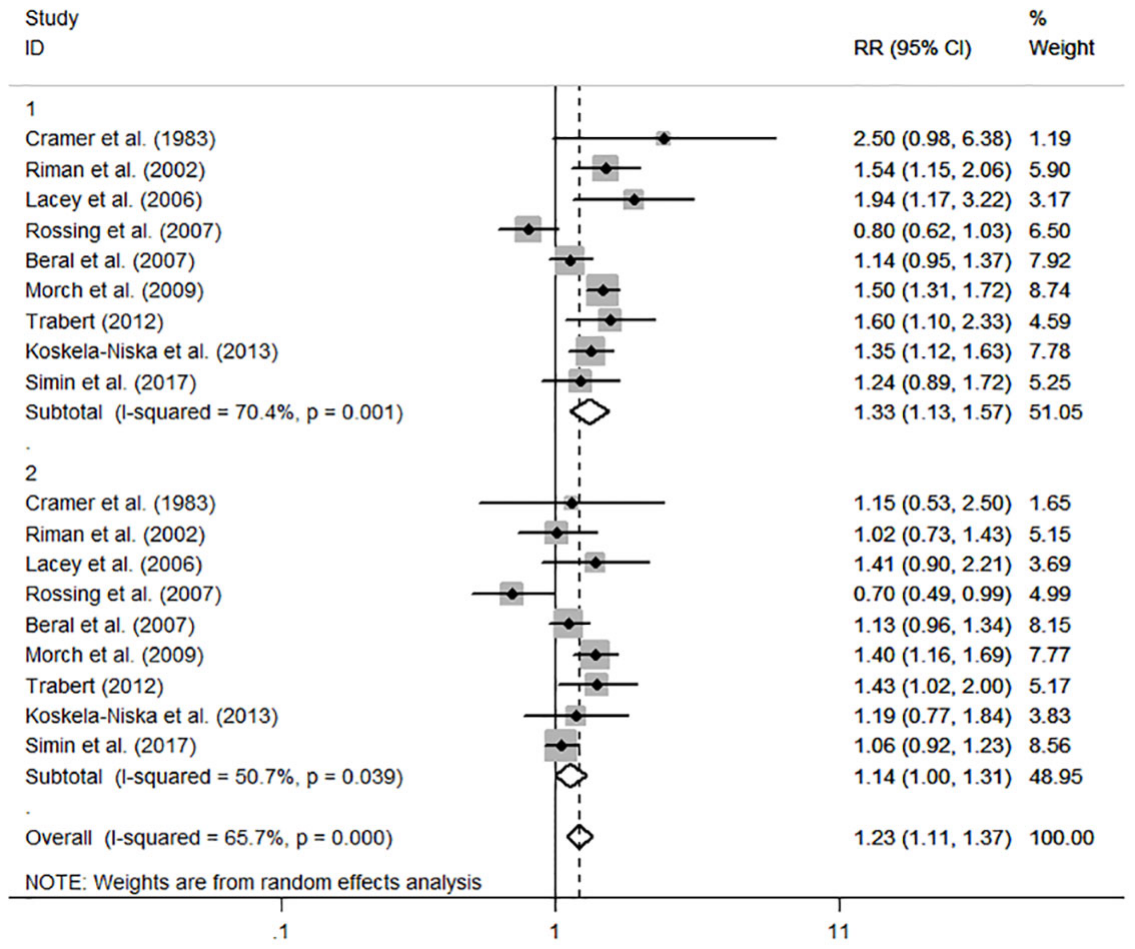
Forest plot of the association between HRT and the risk of ovarian cancer in subgroup analysis stratified by different EPRT regimens. The size of each gray box is proportional to the weight assigned to the respective study, with horizontal lines representing the 95% confidence intervals (CIs).

The individual and summary risk estimates for ovarian cancer with HRT use were presented in **Figures 2**, **3**, classified by different study designs. Cohort studies found a 1.20 (95% CI 1.01–1.44) increased risk in patients with a history of HRT use, while case-control studies showed a 1.13 (95% CI 1.04–1.22) increased risk. The summary result indicated a higher risk in cohort studies. However, after restricting the studies to more recent years, the associated risks became negligible. As shown in **Figures 4**, **5**, the pooled RR of cohort studies conducted after 2010 was 1.15 (95% CI 0.82–1.61), while the pooled OR of case-control studies conducted after 2006 was 1.09 (95% CI 0.93–1.27). Currently, the use of EPRT is more prevalent in HRT than single estrogen to mitigate endometrial stimulation. EPRT can be administered through either continuous or sequential use. As shown in **Table 3**, we examined nine cohort and case-control studies with data on different EPRT regimens and found that result in continuous hormone use (1.14, 95% CI 1.00–1.31) appeared to be similar with sequential use (1.33, 95% CI 1.13–1.57) (**Figure 6**).

**Table 3:** Characteristics of two types of EPRT included in the meta-analysis.

Study	Year	Study region	Hormone type	OR or RR	95% CI
Cramer et al. (34)	1983	USA	EPRT-S	2.50	0.98, 6.38
			EPRT-C	1.15	0.53, 2.50
Riman et al. (49)	2002	Sweden	EPRT-S	1.54	1.15, 2.05
			EPRT-C	1.02	0.73, 1.43
Lacey et al. (8)	2006	USA	EPRT-S	1.94	1.17, 3.22
			EPRT-C	1.41	0.90, 2.22
Beral et al. (4)	2007	United Kingdom	EPRT-S	1.14	0.92, 1.32
			EPRT-C	1.13	0.95, 1.33
Rossing et al. (56)	2007	America	EPRT-S	0.80	0.60, 1.00
			EPRT-C	0.70	0.50, 1.00
Mørch et al. (22)	2009	Denmark	EPRT-S	1.50	1.31, 1.72
			EPRT-C	1.40	1.16, 1.69
Trabert et al. (24)	2012	USA	EPRT-S	1.60	1.10, 2.33
			EPRT-C	1.43	1.03, 2.01
Koskela-Niska et al. (58)	2013	Finland	EPRT-S	1.35	1.12, 1.63
			EPRT-C	1.19	0.77, 1.85
Simin et al. (30)	2017	Sweden	EPRT-S	1.24	0.88, 1.70
			EPRT-C	1.06	0.91, 1.22

RR, relative risk; OR, odds ratio; CI, confidence intervals; EPRT-S, Sequential estrogen + progestin replacement therapy;

EPRT-C, Continuous estrogen + progestin replacement therapy.

### 3.4 Subgroup analyses

In subgroup analysis based on hormone types, we examined the effects of estrogen or estrogen plus progesterone respectively. Among eight cohort studies, we conducted a synthetic calculation and found that both users of ERT (RR=1.29, 95%CI 1.15–1.45) and EPRT (RR=1.25, 95%CI 1.11–1.41) had minor risk of ovarian cancer (**Table 4**). However, in the six case-control studies, the differences were not significant for the ERT (RR=1.34, 95% CI 0.95–1.88) and EPRT (RR=0.95, 95% CI 0.80–1.13) groups (**Table 4**).

**Table 4:** Subgroup satistical results of cohort and case–control studies.

Subgroup Analysis	No. of studies	References	RR/OR (95%CI)	I2 (%)	P Heterogenity
*Cohort studies*					
**hormone type:** ERT use	8	(4, 7, 17, 21, 22, 24, 29, 30)	1.29 (1.15, 1.45)	63.6	0.017
EPRT use	8	(4, 7, 17, 21, 22, 24, 29, 30)	1.25 (1.11, 1.41)	0.0	0.853
**period:** <5y	6	(4, 7, 8, 18, 21, 25)	1.07 (0.96, 1.19)	0.0	0.892
5-9y	7	(4, 7, 8, 18, 21, 24, 25)	1.39 (1.20, 1.62)	35.7	0.156
>=10y	4	(4, 7, 8, 25)	1.52 (1.31, 1.77)	29.9	0.222
**tumor type:** serous	3	(4, 25, 61)	1.57 (1.43, 1.72)	0.0	0.717
endometrioid	3	(4, 25, 61)	1.44 (1.00, 2.06)	71.4	0.030
mucinous	3	(4, 25, 61)	0.66 (0.49, 0.89)	30.3	0.238
clear cell	3	(4, 25, 61)	0.83 (0.61, 1.13)	0.0	0.781
other	2	(4, 25)	1.31 (1.14, 1.51)	0.0	0.649
*Case-control studies*					
**hormone type:** ERT use	6	(10, 43, 52, 54, 56, 62)	1.34 (0.95, 1.88)	98.2	0.000
EPRT use	6	(10, 43, 52, 54, 56, 62)	0.95 (0.80, 1.13)	81.5	0.000
**period:** <5y	11	(10, 34, 38, 41, 42, 49, 50, 54, 56, 58, 62)	1.04 (0.82, 1.33)	79.3	0.000
5-9y	12	(10, 34, 38, 41, 42, 49, 50, 53, 54, 56, 58, 62)	1.13 (0.99, 1.29)	13.7	0.310
>=10y	7	(38, 41, 42, 49, 53, 54, 56)	1.37 (1.02, 1.85)	56.0	0.034
**tumor type:** serous	12	(33, 36, 38, 41–43, 48, 49, 51, 53, 54, 63)	1.17 (1.00, 1.35)	62.1	0.002
endometrioid	12	(33, 36, 38, 41–43, 48, 49, 51, 53, 54, 63)	1.07 (0.81, 1.41)	72.0	0.000
mucinous	11	(33, 36, 38, 41, 43, 48, 49, 51, 53, 54, 63)	0.97 (0.89, 1.05)	0.0	0.729
clear cell	4	(42, 49, 51, 53)	1.26 (0.77, 2.07)	13.8	0.323
other	9	(33, 36, 38, 42, 48, 51, 53, 54, 63)	1.04 (0.84, 1.29)	44.2	0.073

RR, relative risk; OR, odds ratio; CI, confidence intervals; ERT, estrogen replacement therapy; EPRT, estrogen + progestin replacement therapy.

Additionally, the risk was higher for long-term HRT users, especially those using it for more than five years, and even more so for over ten years. In six cohort studies, the summary risk estimates for different durations were 1.07 (95% CI 0.96–1.19) for less than 5 years, 1.39 (95% CI 1.20–1.62) for 5–9 years, and 1.52 (95% CI 1.31–1.77) for more than 10 years. In eleven case-control studies assessing the risk of ovarian cancer for varying hormone durations, the summary risk estimates were 1.04 (95% CI 0.82–1.33) for less than 5 years, 1.13 (95% CI 0.99–1.29) for 5–9 years, and 1.37 (95% CI 1.02–1.85) for more than 10 years. Only the CI for the longest duration crossed 1.0 (**Table 4**).

The influence of HRT varies among different histological subtypes. In cohort studies, there is a significant association between HRT and serous cancer (RR=1.57, 95% CI 1.43–1.72), as well as in case-control studies (OR=1.17, 95% CI 1.00–1.35). Additionally, the use of HRT is linked to an increased incidence of endometrioid cancer in cohort studies (RR=1.44, 95% CI 1.00–2.06) (**Table 4**). This may be due to the fact that endometrial cells are more sensitive to estrogen stimulation. Subgroup analysis results are provided in **Table 4**.

### 3.5 Meta-regression and sensitivity analysis

We conducted a multivariate meta-regression to assess the heterogeneity between the studies included. The heterogeneity was found to be moderate in case-control studies and high in cohort studies, with minimal change even after excluding studies with high RR or OR. The covariates examined were study design, publication year, and study region. However, none of them seemed to have an impact on the between-study heterogeneity.

**Figure 7:**
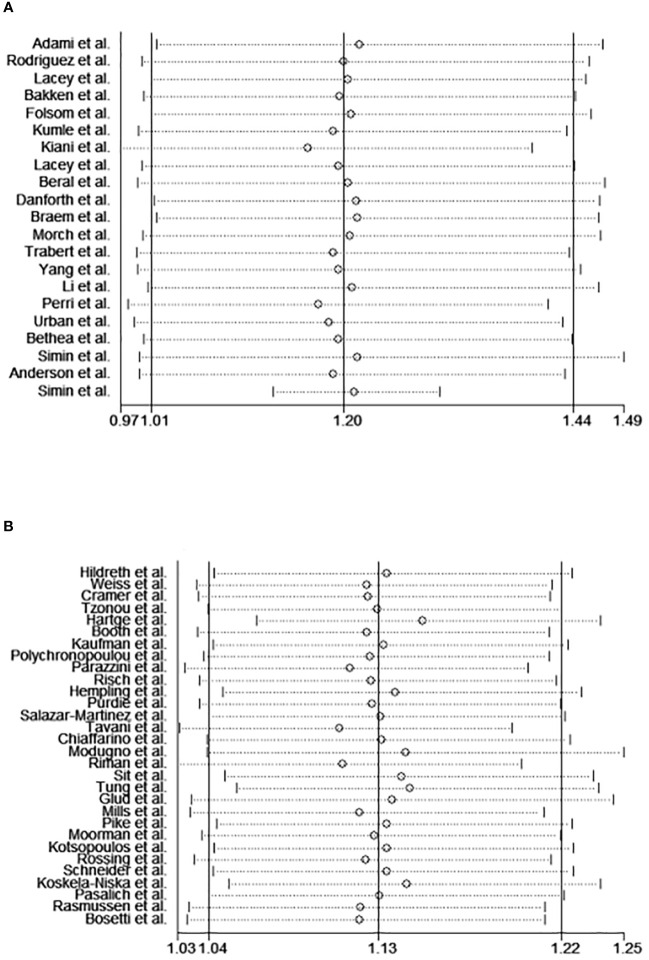
The sensitivity analysis of the meta-analysis of cohort studies **(A)** and case-control studies **(B)**.

Sensitivity analysis was conducted to determine if any particular study had significant implications for the results (**Figures 7A, B**). In cohort studies, the pooled RRs ranged from 1.16 (95%CI 0.97–1.39) to 1.22 (95%CI 1.01–1.47); For case-control studies, the pooled ORs varied from 1.11 (95%CI 1.03–1.19) to 1.15 (95%CI 1.70–1.24). No individual study exhibited this effect.

### 3.6 Publication bias

**Figure 8:**
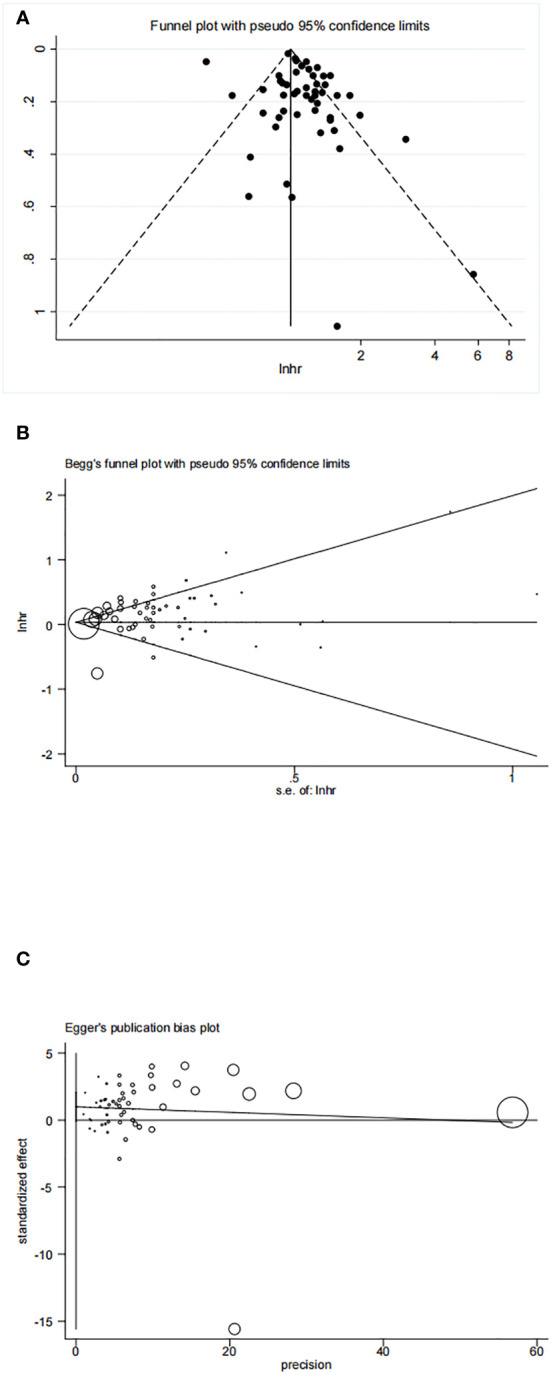
**(A)** Funnel plot of included studies to assess publications bias; **(B)** Begg’s test of included studies to assess publications bias; **(C)** Egger’s test of included studies to assess publications bias.

The funnel plot, Begg’s test, and Egger’s test results are displayed in **Figures 8A–C**. These tests indicated no significant publication bias (Begg’s test P=0.407, Egger’s test P=0.070) in our included articles on the association between HRT and ovarian cancer risk.

## 4 Discussion

Previous Meta-analysis have yielded conflicting findings regarding the association between hormone use and ovarian cancer risk. Two studies found no association during the early period (15, 16), while another article published in 1998 reported positive findings (15), but both used the fixed effects model and encountered heterogeneity. In a recent article, the authors suggested a summary risk estimate of 1.29 (95%CI 1.11–1.38) for menopausal HRT. Its results were positive, as the authors selectively highlighted the most significant findings from each included study (60). A meta-analysis of 52 epidemiological studies since 1970 revealed that women initiating hormone therapy for 5 years around age 50 experience approximately one additional case of ovarian cancer per 1000 users (64). In 2016, The International Menopause Society (IMS) noted that the relationship between HRT and ovarian cancer remains unclear. The risks and benefits of hormone therapy varied between women undergoing menopausal transition and older women (65). In 2023, IMS published a practitioner’s toolkit for managing menopause. They suggested thorough evaluation and individually tailored drug regimens to ensure appropriate care for patients (66). The European Menopause and Andropause Society (EMAS) and North American Menopause Society (NAMS) have insufficient evidence linking HRT to ovarian cancer. However, EMAS advises caution regarding hormone therapy in women with serous epithelial ovarian cancer. NAMS emphasizes a level II finding indicating a slight but statistically significant ovarian cancer risk associated with hormone use in observational studies (3, 67).

In this meta-analysis, we aimed to assess the relationship between hormone use and ovarian cancer risk. Our key findings are as follows:

First, previously hormone use increased the risk of ovarian cancer in both cohort studies and case-control studies. However, these effects became trivial when we limited the study period to recent years. These findings imply that advancements in medication and adjustments in administration methods have potentially reduced the risk of HRT on ovarian cancer. Due to the limited number of studies from the past decade included in this research, the results are subject to certain limitations. It is conceivable that the influence of HRT on the incidence of ovarian cancer is declining.

Second, in subgroup analysis of hormone type, only cohort studies manifested that the use of either single estrogen or estrogen plus progesterone use could increase the risk of ovarian cancer, but this finding was not significant in case-control studies. In the WHI trial, Anderson et al. reported a non-significant hazard ratio of 1.64 in EPRT users (5). However, they did not compare the risk between ERT and EPRT, and there was no data available on estrogen alone (68). Some other studies indicated that EPRT had a lower ovarian cancer risk compared to ERT in HRT users (69, 70). This aligns with the findings of our research. The hormone types were classified in 8 cohort studies and 6 case-control studies. The risk of estrogen used alone was higher than when estrogen and progesterone were used together, both in cohort and case-control studies. Our research includes more articles on ERT and EPRT to reinforce this conclusion. Estrogen receptors are present on the surface of both normal ovaries and malignant ovarian tumors (61). The use of HRT in ovarian cancer carries a risk due to its estrogen element. The process of ovulation, including rupture and repair, can stimulate the oncogenesis of epithelial ovarian cells. Oral contraceptives containing both estrogen and progesterone have been shown to reduce the risk of ovarian cancer. Progesterone plays a role in counteracting the effects of estrogen in the proliferation of ovarian cells and can inhibit ovulation through negative feedback on the Hypothalamus-Pituitary-Ovary (H-P-O) axis during menstruation. However, this effect is not present after menopause (61, 62). Estrogens can act through estrogen receptors to regulate various cellular processes in ovarian cancer cells, including proliferation, epithelial-mesenchymal transition (EMT), invasiveness, differentiation, and inflammation (63), while progesterone and its receptor play an anti-tumor role in the development of ovarian cancer (71). Further research is needed to understand the concrete mechanisms of these hormones.

Previous studies have shown that both continuous and sequential hormone therapy (HT) are associated with an increased risk of ovarian cancer. However, these studies did not compare the two types of therapy (72). In our analysis, we found that continuous hormone use had a similar risk of ovarian cancer compared to sequential use. Continuous hormone use involves taking both estrogen and progesterone every day, similar to oral contraceptives. Whether this type of therapy can also protect ovarian cells from malignant transformation is still unknown. Interestingly, all the studies that differentiated EPRT users into continuous and sequential groups suggested that the former group has a lower risk of ovarian cancer. Considering that continuous hormone use with estrogen and progesterone, like oral contraceptives, has a lower cancer risk and sequential EPRT doesn’t have any major advantages, we recommend continuous EPRT treatment as the first choice for those long-time users experiencing perimenopausal syndrome, who do not prioritize their menstruation.

In addition, the overall risk of ovarian cancer did not increase for nonusers who used hormones for less than five years. In our study, we analyzed 11 case-control studies that provided data on long-term HRT use. The risk of using hormones for more than 5 years and 10 years was calculated, resulting in a summary risk of 1.13 (95%CI 0.99–1.29) and 1.37 (95%CI 1.02–1.85) respectively. Additionally, six cohort studies provided risk values for different durations. They revealed a significant risk for users who had been taking hormones for more than five years (RR=1.39, 95%CI 1.20–1.62), with an even higher risk for those exceeding 10 years of usage (RR=1.52, 95%CI 1.31–1.77). This result suggests that the risk of ovarian cancer increases as the duration of HRT use extends. Further evidence is required to support the recommendation of avoiding steroid hormone usage for more than ten years.

At last, in the analysis of histologic subgroups, we observed that serous cancer was more susceptible than other cancer types in both types of research. The cohort studies’ analysis revealed a significant increase in ovarian endometrioid cancer risk with HRT use (RR=1.44 95%CI=1.00, 2.06). For the low incidence of mucinous and clear cell carcinoma, the evidence is not very convincing.

It is necessary to evaluate the heterogeneity between-studies in meta-analysis. Moderate heterogeneity was observed in case-control studies, while cohort studies showed high heterogeneity. However, the published year and study region didn’t contribute to the heterogeneity, as determined by meta-regression analysis. The between-study heterogeneity did not significantly decrease after excluding several studies with remarkably increased or decreased RR values. And there was no significant impact on the results. Therefore, these findings can be considered reliable.

There are some advantages in our study. Firstly, we obtained more accurate and convincing results due to the sufficient data and sample size compared to previous studies. In addition, we extracted ORs and RRs from the original studies encompassing all participants to offer a comprehensive assessment of the risk associated with both all HRT users and recent HRT users. These findings suggest that modern HRT regimens are becoming safer. This can serve as a valuable reference for those considering menopausal HRT. Thirdly, for EPRT users who do not care about menstruation, the continuous pattern could be preferable to the sequential pattern. But this advantages of decreasing the ovarian cancer risk may not that prominant. Furthermore, our conclusion highlights that long-term use of HRT for more than 10 years is associated with a significantly higher risk of ovarian cancer. Moreover, the subgroup analysis revealed a strong relationship between HRT use and serous ovarian cancer, while cohort studies also indicated a higher risk of endometrioid cancer among HRT users. Finally, between-study heterogeneity and sensitivity analyses confirm the stability of our conclusions.

Some limitations exist in our study. Firstly, the study groups and adjusted confounders differ in each research, which may partially affect the results due to these biases. The insufficient follow-up period of some researchers would also miss some potential cases. Additionally, the therapeutic regimen of HRT has evolved over time. In the past, estrogen was commonly used alone to treat the menopausal syndrome. However, nowadays, it is preferred to prescribe a combination of estrogen and progesterone. In this meta-analysis, we prioritize selecting the data on estrogen plus progesterone hormone usage if it is described in the articles. In some studies, there is insufficient accurate data for different types of hormone use, so the summary data of OR or RR represented all hormone users. Thirdly, the relationship between HRT and histologic subtypes lacks strong evidence due to limited data. However, long-term use of HRT has consistently shown higher rates of ovarian cancer in multiple studies. At last, it is important to acknowledge that each study may have inherent biases that could influence the results. And it is also worth noting that positive results are more likely to be published, while negative findings may be overlooked in the literature search process.

## 5 Conclusion

In conclusion, our findings suggest that the use of HRT can increase ovarian cancer risk in certain cases. However, when we restricted the study period to the past decade, the associated risk was minimal. Considering the benefits of HRT in managing menopausal symptoms, such as preventing osteoporosis, thromboembolic disease, and climacteric disease, it has a wide range of applications. Individualized prescription of different types of HRT treatments and strict follow-up are crucial in preventing the potential side effects of tumors.

## Data availability statement

The original contributions presented in the study are included in the article/supplementary material. Further inquiries can be directed to the corresponding author.

## Author contributions

HX: Writing – original draft, Software, Formal analysis, Data curation. LW: Writing – original draft, Data curation, Conceptualization. LS: Writing – review & editing, Resources, Investigation. SX: Writing – review & editing, Software, Resources, Funding acquisition.
